# Effect of genome composition and codon bias on infectious bronchitis virus evolution and adaptation to target tissues

**DOI:** 10.1186/s12864-021-07559-5

**Published:** 2021-04-07

**Authors:** Giovanni Franzo, Claudia Maria Tucciarone, Matteo Legnardi, Mattia Cecchinato

**Affiliations:** Microbiology and Infectious Diseases, Department of Animal Medicine, Production and Health (MAPS), University of Padua, Viale dell’Università 16 - 35020 Legnaro, Padua, Italy

**Keywords:** Infectious bronchitis virus, Codon Bias, Genome composition, Evolution

## Abstract

**Background:**

Infectious bronchitis virus (IBV) is one of the most relevant viruses affecting the poultry industry, and several studies have investigated the factors involved in its biological cycle and evolution. However, very few of those studies focused on the effect of genome composition and the codon bias of different IBV proteins, despite the remarkable increase in available complete genomes. In the present study, all IBV complete genomes were downloaded (*n* = 383), and several statistics representative of genome composition and codon bias were calculated for each protein-coding sequence, including but not limited to, the nucleotide odds ratio, relative synonymous codon usage and effective number of codons. Additionally, viral codon usage was compared to host codon usage based on a collection of highly expressed genes in IBV target and nontarget tissues.

**Results:**

The results obtained demonstrated a significant difference among structural, non-structural and accessory proteins, especially regarding dinucleotide composition, which appears under strong selective forces. In particular, some dinucleotide pairs, such as CpG, a probable target of the host innate immune response, are underrepresented in genes coding for pp1a, pp1ab, S and N. Although genome composition and dinucleotide bias appear to affect codon usage, additional selective forces may act directly on codon bias. Variability in relative synonymous codon usage and effective number of codons was found for different proteins, with structural proteins and polyproteins being more adapted to the codon bias of host target tissues. In contrast, accessory proteins had a more biased codon usage (i.e., lower number of preferred codons), which might contribute to the regulation of their expression level and timing throughout the cell cycle.

**Conclusions:**

The present study confirms the existence of selective forces acting directly on the genome and not only indirectly through phenotype selection. This evidence might help understanding IBV biology and in developing attenuated strains without affecting the protein phenotype and therefore immunogenicity.

**Supplementary Information:**

The online version contains supplementary material available at 10.1186/s12864-021-07559-5.

## Background

Infectious bronchitis virus (IBV), a member of the family *Coronaviridae,* genus *Coronavirus,* classified within the species *Avian coronavirus* (https://talk.ictvonline.org/), is one of the most relevant viral poultry pathogens and responsible for remarkable economic losses worldwide due to both direct and indirect costs [1]⁠. IBV mainly causes upper respiratory tract disease, which can lead to high mortality when secondary infections occur. High mortality is also associated with some strains able to cause nephritis. Additionally, the genital tract of layer and breeder birds can be affected, causing reproductive disorders and altered egg production [2]⁠.

IBV is characterized by a single-stranded positive-sense genome of approximately 27 kb that codes for at least 10 open reading frames (ORFs) [1]⁠. The 5′ two-thirds of the genome encodes two polyproteins, pp1a and pp1ab, which are then proteolytically cleaved in 15 nonstructural proteins. Production of pp1ab requires the translating ribosome to change the reading frame at the frameshift signal that bridges ORF1a and ORF1ab [3]⁠.

The rest of the genome encodes structural proteins, including Spike (S), Envelope (E), Matrix (M) and Nucleocapsid (N) [1]⁠. Accessory proteins (3a, 3b, 5a and 5b) not fundamental for virus replication [4] have been identified and proven to be involved in virus–host interactions and immune response modulation during infection [5]. Coronaviruses are well-known to interact at various levels with cell signalling and innate and adaptative responses to maximize their replicative success and limit recognition by the host defence system [6, 7]. Although most of the current knowledge is based on experimental evidence, the increasing sequencing capability, coupled with improved modelling approaches, has contributed in several ways to the study of these viruses. Indeed, sequence analysis has allowed us to reconstruct the epidemiology of IBV strains, identify their differences, estimate the causes and strength of selective pressures shaping their evolution and evaluate the consequences, just to mention a few [8–10]⁠. However, with limited exceptions, genome analysis has been considered an indirect and easier way to investigate IBV protein features. Nevertheless, it must be stressed that the viral RNA genome cannot be reduced to the genotype concept (i.e., a mere “string of text” coding for a certain phenotype), as the RNA molecule has its own phenotypic features and is thus under the action of direct selective pressures. For example, genome base composition can alter physical properties, such as stability at different temperatures, pH, and metal concentration [11–13]⁠, as well as functional aspects, such as those ascribable to the presence of secondary structures. Several studies have demonstrated the presence of a relevant genomic signature in dinucleotide frequencies in different organisms. In eukaryotic genomes, TpA is broadly under-represented, likely because of the higher susceptibility to degradation by ribonucleases, lower thermal stability and occurrence of the TA dinucleotide in two stop codons as well as in many regulatory regions [14, 15]⁠. In addition, the CpG dinucleotide is similarly underrepresented because cytosine in CG dinucleotides is easily methylated, and this form tends to spontaneously deaminate to thymine [16]⁠.

Interestingly, even the microbiota of different environments features distinct patterns, supporting the direct or indirect effect of environmental conditions on organism genome composition [17]⁠.

Codon bias is another phenomenon potentially affecting organism fitness in the absence of a direct effect on protein primary structure. Because of the degeneracy of the genetic code, the 20 amino acids are encoded by 61 codons. As there are more codons than amino acids, the genetic code is necessarily redundant, and most amino acids are encoded by two to six different codons [16]⁠. However, different synonymous codons are used with different frequencies among organisms or even among tissues of the same organism [18, 19].

Two non-conflicting hypotheses have been proposed to justify codon bias occurrence: 1) the mutational hypothesis suggests that uneven codon usage is due to the underlying genome composition and therefore to forces favouring certain types of mutations [20]⁠; 2) the selectionist hypothesis postulates the occurrence of selective forces directly acting on codon bias. In fact, a positive correlation has been observed between gene expression and codon bias, with highly expressed genes enriched in the most frequent optimal codons. In addition to translation efficiency, codon usage has been related to gene expression level, translation fidelity, appropriate protein folding and overall organism fitness [16, 21, 22]⁠.

Currently, the most accepted model, the mutation-selection-drift balance model of codon bias, proposes selective forces favouring preferred codons, whereas mutation pressure and genetic drift allow for the persistence of minor ones [23, 24]⁠.

Although the intensity of selective forces acting on codon bias are often considered weak [16]⁠, viruses can represent a remarkable exception. As intracellular obligate parasites, they must accomplish two fundamental tasks: escaping from the host immune system and being able to efficiently exploit the cell synthetic machinery. Accordingly, the virus-host association in terms of codon bias and genome composition has been reported by different authors [25–28]⁠, and in some instances, progressive viral adaptation after a host jump has been proven [28, 29].

These viral features can clearly affect IBV biology, fitness and virulence, although the issue has rarely been investigated [30]⁠, despite the availability of a remarkable number of complete genomes and host tissue-specific gene expression levels.

## Results

### Genome base composition

Overall, IBV coding regions showed a lower percentage of C and G nucleotide, although with a certain variability among proteins. When the distribution was evaluated for different codon positions, the CG content decreased from the first to the third codon position. A summary of genome composition features is provided in Additional file 1 and Additional file 2.

**Fig. 1:**
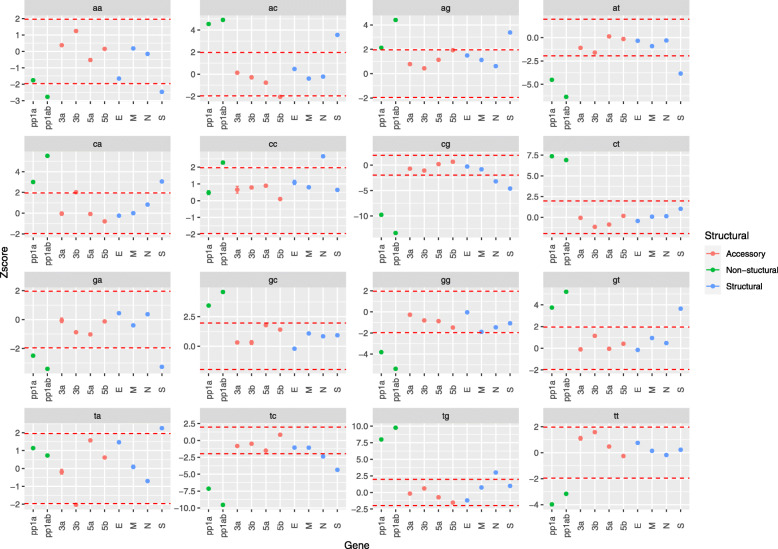
Mean (point) and 95% confidence interval (errorbar) calculated for the Z-score each dinucleotide-gene pair. The dashed lines (i.e. Z-score ± 1.96) highlight the cut-off for significantly under- and overrepresented dinucleotides. Structural, non-structural and accessory proteins have been color-coded. The figure was generated using ggplot2 [32]

Dinucleotide pairs *Rho* statistic calculation analysis revealed that several residues could be considered as over- or underrepresented (Additional file 3) according to the cut-offs proposed by Karlin et al., (1998) [31]. However, the limited sequence length and the likely confounding effect of codon bias and amino acid sequence suggest caution in the results interpretation. The Z-score calculated by random permutation of synonymous codons represents thus a more robust estimation. This statistic confirmed the presence of different dinucleotide pairs significantly over or under-represented compared to what is expected by chance. Particularly, CpG and TpC were highly under-represented in pp1a and pp1ab and to a lesser extent in the two main structural proteins, S and N. Accessory, M and E proteins were within the expected ranges. Similar patterns were observed for ApT and GpA in the pp1a, pp1ab and S. On the contrary, pp1a, pp1ab and S revealed over-represented ApC, ApG, CpA, GpT and TpG dinucleotide pairs. CpT and GpC were overrepresented in polyprotein region only (Fig. 1). Overall, accessory, E and M proteins had a dinucleotide content essentially explainable by C and G frequency only.

The 2 principal components of PCA performed on Z-score explained almost 80% of the overall variability, and were therefore used to summarize the dinucleotide features of IBV genes.

**Fig. 2:**
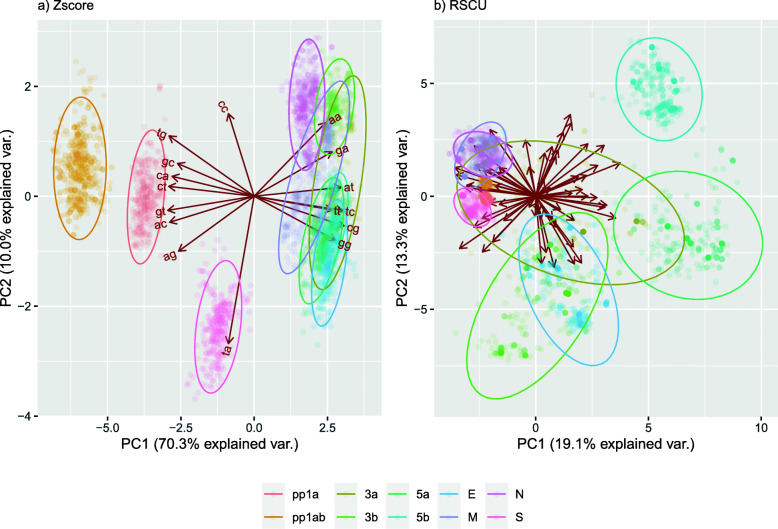
Scatter plot based on the first two components of the PCA performed on Z-score (**a**) and RSCU (**b**) calculated for all IBV proteins (color-coded). The PCA loadings are represented as arrows. However, for graphical reasons the labels have been removed from (**b**) and the loading values are provided in Additional file 4. The 95% confidence ellipses around clusters are also reported. The figure was generated using ggplot2 [32]

Two different patterns were clearly observed. pp1a, pp1ab and S protein formed separate clusters on the negative side of PC1, while the rest of the proteins constituted a more homogeneous group, being the accessory proteins located on the positive extreme of PC1. M and N proteins, featured by less positive values, were differentiated based on PC2 values. Similarly, 5a and 5b were differentiated from 3b through PC2 scores, although a sparser distribution and relevant overlapping were present, involving especially protein 3a (Fig. 2a). Principal components loading analysis confirmed the high weight of several nucleotide pairs in differentiating the two main gene groups along the PC1 (e.g.. CpG, TpC, ApT, etc. were positively correlated to PC1), while TpA and CpC were especially correlated with PC2 scores (Fig. 2a).

### Relative synonymous codon usage

**Fig. 3:**
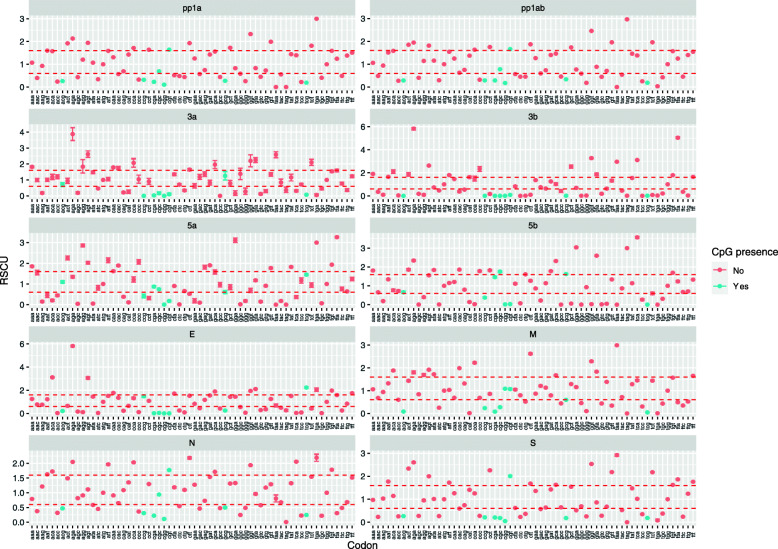
Mean (point) and 95% confidence interval (errorbar) of RSCU statistic calculated for each gene−codon pair. When the CpG pair was present in the codon, it has been highlighted in blue. The dashed lines (i.e. RSCU = 0.6 and 1.6) highlight the cut-off for significantly under- and overrepresented codons, respectively. The figure was generated using ggplot2 [32]

Relevant differences in RSCU were observed among codons, similarly to what observed for dinucleotide frequency. Although a certain variability was observed among proteins, some common patterns could be observed. Particularly, codon containing the CpG dinucleotide were within the expected ranges or, more frequently, under-represented (Fig. 3). Codon CGT and CGC were the only exceptions, being the former slightly overrepresented in 1a, 1ab, N and S genes and the latter in 5b one (Fig. 3). Based on PCA eigenvalues evaluation, the first two principal components (PC1 and PC2) were maintained since explaining more than 30% of the overall variability. The observed pattern was featured by a higher similarity in codon bias usage among structural and non-structural proteins compared to accessory ones (Fig. 2b). Particularly, a closer relationship was observed between pp1a, pp1ab and S protein, and between M and N ones. The E protein was the only exception, forming a separated cluster largely overlapping with the codon usage pattern of 3a and 3b proteins, which had a highly heterogeneous distribution. Although comparably heterogeneous, 5a and 5b formed essentially independent groups. PCA loading analysis highlighted the primary contribution of CpG enriched/depleted codon in determining PC1 values (being 6 out of 8 positively correlated to PC1) (Additional file 4). Similarly, 6 out of 8 of CpG enriched codons contributed positively to the PC2. In both instances, the CpG demonstrated higher loadings on average compared to the other codons. However, the limited number of CpG rich codons prevented any robust statistical inference. Therefore, structural and non-structural proteins were located in PCA regions representing codons with low CpG content, i.e. negative values on PC1 (pp1a, pp1ab, M, S and N) or PC2 (E).

### Nc and Nc plot

**Fig. 4:**
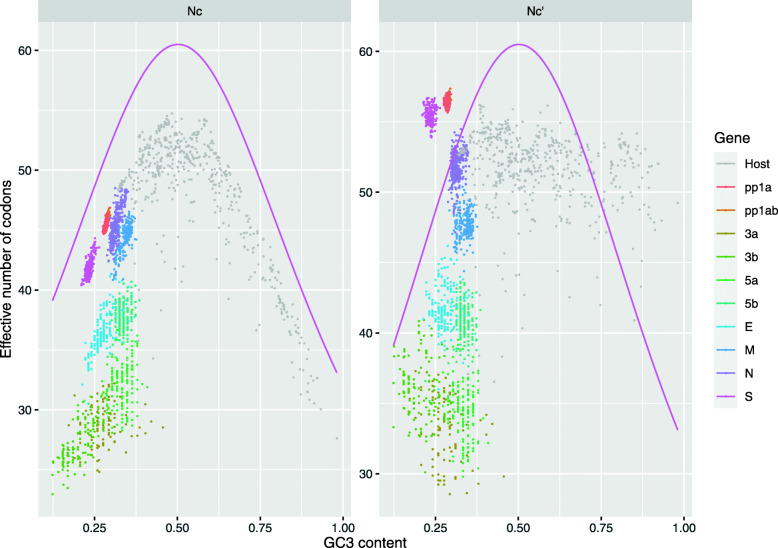
Scatterplot reporting the relationship between Nc and Ncp and GC3 content of IBV coding sequences. IBV proteins have been color−coded while the host genes have been reported in grey. The line representing the expected Nc values, which would result from GC composition being the only factor influencing the codon usage bias, has been superimposed. The figure was generated using ggplot2 [32]

Effective number of codon calculation revealed a relevant difference among IBV proteins. Accessory proteins showed the more biased codon usage, with lower Nc values compared to structural and non-structural ones (Additional files 1 and 5). When nucleotide composition was accounted for, higher Nc’ values were obtained. However, the above-mentioned difference remained or was even magnified (Fig. 4).

The Nc values were constantly lower than the ones expected based on CG3 content only. While this remained true for accessory, E and M protein coding regions even after accounting for genome composition, the Nc’ of the N gene lied on the expected value and was higher for the polyproteins and S genes, which overall showed values comparable with the host ones (Fig. 4).

### Neutrality plot, general average hydropathicity (gravy) and aromaticity (aroma) indices

A significant association (*p* < 0.05) between GC12 and CG3 content was demonstrated for pp1a (b = 0.10), 3a (b = 0.10), 5b (b = 0.17), E (b = 0.16), M (b = 0.15), S (b = 0.11) and N (b = 0.09) genes. Therefore, mutation drift accounted for approximately 10% of the codon bias of 1a, 3a, S and N genes, while a more intense effect (approximatively 15–20%) was estimated for 5b, E and M ones. Overall, the impact of mutation bias can be considered low. Similarly, regression analysis demonstrated that Gravy and Aroma indices were significantly associated (p < 0.05) with the PC1 and/or PC2 of Z-score and/or RSCU (Additional file 6), a trend confirming the occurrence of additional selective pressure acting on codon and dinucleotide composition rather than the effect of genome composition or mutation bias only.

### CAI analysis

**Fig. 5:**
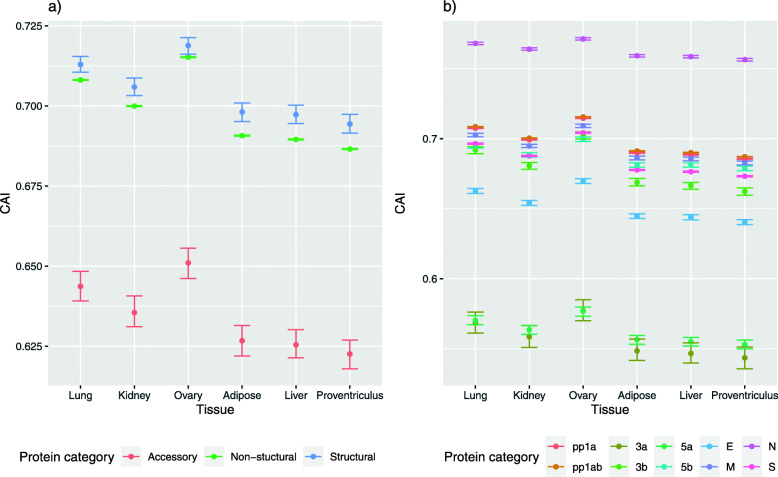
Mean (point) and 95% confidence interval (errorbar) of the CAI index calculated for genes corresponding to different protein category (**a**) and proteins (**b**) (color-coded) with respect to different host tissues. The figure was generated using ggplot2 [33]

The CAI of IBV proteins was calculated based on the relative adaptiveness of each codon based on the most expressed genes of considered tissues. Irrespectively of the considered organ, the CAI was on average lower for accessory proteins compared to non-structural and especially structural ones (Fig. 5a). However, when single genes were evaluated, a more complex pattern was observed. Most genes had a value of approximate 0.7, N showed the higher value while accessory protein 3a and 3b had the lowest CAI value. E gene was the structural protein coding gene with the lowest CAI value (Fig. 5b and Additional file 1). Despite these differences, a constantly lower CAI was observed in non-target tissues compared to target ones.

## Discussion

The present study highlights a relevant heterogeneity in genome composition and codon bias among IBV genes. Different dinucleotide pairs were shown to be significantly underrepresented, as demonstrated by several dinucleotide odds ratio values lower than the 0.78 and 1.23 cut-offs proposed by Karlin et al. (1998) [31] (Additional file 3). However, these thresholds can be considered accurate for long sequences only [31]⁠. Additionally, dinucleotide frequency might be affected by codon bias and by amino acid composition imposed by protein functional constraints. After accounting for the codon bias and amino acid constraints of the studied sequences using a permutation approach, several dinucleotide pairs still significantly deviated from what was expected by chance alone (Fig. 1). Similar to what has been described for influenza A virus (IAV) [34]⁠, noteworthy variability was observed among IBV genes. In particular, the CpG pair was highly underrepresented in the genes encoding polyprotein, spike and nucleocapsid. This pair is well known to be underrepresented in eukaryotic genomes, as cytosine in CG dinucleotides are easily methylated and tend to spontaneously deaminate to thymine [15, 16]. However, methylation does not seem to occur in viruses, especially in RNA viruses that use their own synthetic apparatus for genome replication and transcription [35]⁠. Other causes should thus be evaluated. Unmethylated DNA is a well-known target of the pattern recognition receptor (PRR) Toll-like receptor 9 (TLR-9) in mammals and is thus involved in innate immune response activation. Interestingly, TLR-9 is absent from the avian genome, and no orthologue gene has been identified [36, 37]⁠⁠. Nevertheless, chicken TLR-21 has a comparable function [38]⁠, despite some differences in activation when stimulated by pathogens [39, 40]⁠. Therefore, the tendency of DNA viruses to reduce their CpG content can be easily explained. Much more under debate is whether similar forces act on RNA viruses. Other TLRs, such as TLR3, TLR7, and TLR8 (which is a pseudogene in chickens), PRRs such as RIG-I (absent in chickens) and MDA5 have been demonstrated to target RNA viruses [41, 42]⁠, but none has been proven to recognize CpG regions. Nevertheless, more recent evidence suggests that ssRNA oligonucleotides expressing unmethylated CpG can elicit monocytes and stimulate PBMCs through a mechanism independent of TLR3, − 7, − 8 or − 9 [43]⁠.

Atkinson and colleagues demonstrated that experimentally increasing the CpG and, to a lesser extent, the TpA content leads to echovirus 7 attenuation, lower replication rates and low competitive fitness relative to wild-type [44]⁠. More recently, Takata et al. (2017) proved that the zinc-finger antiviral protein (ZAP) selectively binds to sequences containing CpG dinucleotide and that HIV strains with a modified CpG content are defective in normal cells but able to replicate in ZAP-defective cells [45]⁠.

Therefore, host immune pressure can be considered the most likely selective force shaping IBV genome towards a reduction in CpG motifs, as proposed for other viruses [46, 47]⁠. In contrast to influenza, TpA was not underrepresented in any of the viral proteins, similar to what was previously reported for other members of *Coronaviridae* [48]⁠. This evidence was unexpected, as TpA upregulation had detrimental effects on viral fitness according to Atkinson et al. (2014) [44]. Thus, other host response mechanisms might be involved and potentially circumvented in various ways by viruses belonging to different families.

Interestingly, the polyproteins and spike protein exhibited the most biased dinucleotide usage and were clearly differentiated from the others in PCA (Fig. 2). A lower variability, suggestive of stronger constraints, was also evidenced, especially when compared to accessory proteins. Two phenomena might contribute to the observed scenario. The first involves a higher transcription level and mRNA abundance of genomic regions coding for abundant viral proteins (S) or functional ones (pp1a and pp1ab). Additionally, a large number of genomic RNAs (constituted for two-thirds by the polyprotein coding region) are produced and present in the cytoplasm before encapsidation. RNA abundance might represent a factor imposing the minimization of immune-stimulatory domains such as CpG ones. Regardless, transcription of these genes appears to be comparable to that of other IBV proteins [49]⁠. An alternative hypothesis involves the absolute number of CpG molecules. Significantly, these proteins are the longest ones, and a significant negative correlation was found between the CpG Z-score and IBV CDS length (b = − 0.002; *p* < 0.001). Therefore, the higher absolute CpG content in mRNA molecules might reduce the relative amount, minimizing viral recognition. Interestingly, the relationship between the CpG absolute count and CDS length can be adequately described by two ratios: one representative of pp1a, pp1ab and S and another, higher one, of the remaining CDS (Additional file 7). Therefore, additional forces are to a certain extent, likely contributing to the observed dinucleotide composition.

The evaluation of codon bias based on PCA allowed us to classify IBV proteins, albeit less clearly. In particular, pp1a, pp1ab and S had a significantly overlapping distribution, closely mimicked by the N and M proteins. On the other hand, E and accessory proteins demonstrated a different and much sparser distribution (Fig. 2). It could be speculated that these differences are linked to differential gene expression and that proteins expressed at similar levels, especially interacting ones, tend to have correlated levels of codon bias [16, 50]⁠. CAI evaluation supported the proposed hypothesis. Overall, IBV codon bias appears to be more adapted to that of target tissues than non-target tissues, including other epithelial and *parenchymatous* organs (Fig. 5). Additionally, higher CAI was observed for the nucleocapsid protein, which is an abundant structural protein, followed by other structural and non-structural proteins vital for viral replication and whose concerted interaction is necessary for viral encapsidation and infectivity [51, 52]⁠. These results, although based on a broader database and evaluated in a tissue-specific fashion, are largely in agreement with the evidence obtained by Brandao et al. (2013) [53]⁠, strengthening their robustness. In general, we observed evidence for selective forces optimizing codon profiles, especially for genes coding for highly expressed proteins and fundamental for viral viability. Accessory proteins were also featured by a higher heterogeneity in RSCU distribution (Fig. 2b), which appears to involve lower constraints in their codon usage bias. Nevertheless, the shorter length of their coding region might also have magnified the effect of single, random mutations, thus increasing the “background noise” and affecting the observed variability.

The effective number of codon estimations confirmed the opposite tendency of these groups of proteins. The overall Nc was consistently lower for accessory proteins, indicating to a more biased use of synonymous codons (Fig. 4 and Additional file 5). The genome composition, although relevant, cannot fully explain this pattern. In fact, even after accounting for this confounding factor, most of the genes still deviate significantly from the expected values based on GC3 content only, confirming the action of additional pressures other than mutation bias, in agreement with neutrality plot results. Features allowing viral strains to mimic the genome composition and codon bias of the host or tissues where they replicate can be expected to be under strong selective pressures. Moreover, the huge sample size of viral populations within the same host or cell can favour natural selection over genetic drift, even in the presence of modest selective coefficients.

The Gravy and Aroma significant correlation with PC representative of dinucleotide and Codon composition suggests that also selective pressures acting on proteins could indirectly affect these viral features. Interestingly, the previously reported differences among structural, non-structural and accessory proteins do not seem to hold for Gravy and Aroma indices (Additional file 6). Therefore, differential selective patterns can be suggested to act on viral genome and proteins, although both can indirectly affect the nucleotide and codon composition.

In particular, the polyprotein, S and N coding genes showed a lower bias compared to what was expected by chance alone and mimicked the effective number of codons used by the host (Fig. 4). In contrast, accessory proteins exhibited a much more restricted codon usage (and therefore lower adaptation) than chicken proteins. It could therefore be proposed that structural and non-structural proteins exhibit a broad codon spectrum (i.e., lower codon bias), more similar to the host, to overcome potential replication restriction due to the limiting effect of rare tRNAs, which can induce long waiting times and stall elongation [15]⁠.

However, the results of a recent study evaluating IBV gene expression by ribosome profiling appear to reject this hypothesis: despite being highly transcribed, the translation efficacy of the polyprotein, S and N genes was reported to be lower than that of other proteins, including accessory proteins [49]⁠. A limited role of codon bias in gene expression regulation thus cannot be excluded [54]⁠. Our study demonstrates that codon bias is highly affected by overall nucleotide composition, particularly by dinucleotide frequency. Therefore, it is likely that the selection apparently acting on codon bias is largely ascribable to the underlying selection aiming to minimize CpG content and limit viral recognition [34]⁠. Regardless, the remaining differences in terms of codon bias, ENC and CAI among proteins with similar dinucleotide patterns and the different adaptations to target and non-target tissues can hardly be justified by dinucleotide composition alone. Consequently, an actual effect of codon bias on viral fitness can likely be claimed, despite apparently conflicting with experimental evidence [49]⁠.

Although extremely accurate, the study of Dinan et al. (2019) [49] analysed IBV gene expression in chicken kidney primary cell culture, which likely does not represent the actual cell biology in vivo. Additionally, only a “snapshot” of IBV and cell gene expression was obtained, corresponding to a particular moment of the viral cell cycle. Nevertheless, cell transcription activity and pathways can change remarkably at different cycle stages, and differential tRNA abundance can influence viral RNA protein synthesis [55–57]⁠⁠ Continuously expressed proteins such as structural ones and those critical for viral replication might benefit from a lower codon bias, be more adapted to the codon spectrum used by target tissue cells and less susceptible to the variation in tRNAs throughout the cell cycle. The high codon bias of some proteins, accessory ones in particular, might contribute to the regulation of expression of these proteins, favouring their presence in particular cell phases. Additional and more focused experimental studies should be performed to evaluate this theoretically plausible hypothesis.

## Conclusions

Overall, the present study demonstrates that different forces shape the IBV genome and coding sequences in addition to those acting at the protein level [9]⁠. Constraints in dinucleotide frequency reducing viral recognition by innate immune response likely play both a direct role, conditioning genome composition, and an indirect role, affecting codon bias. However, several lines of evidence support the presence of residual selection acting directly on codon usage, which appears to be linked to host tissue adaptation and potentiality in the regulation of individual protein expression.

This evidence might help understanding IBV biology and the development of attenuated strains without affecting the protein phenotype and therefore immunogenicity. Dedicated experimental studies, based on reverse genetics also, could be of remarkable benefit in confirming the association between viral fitness indexes and codon bias or dinucleotide composition.

## Methods

### Dataset

The whole collection of IBV complete or almost complete (including all coding regions) genomes (*n* = 383) was downloaded from Genbank (accessed 28/03/2020). In-house developed Python scripts were used for gene and feature extraction, using the Biopython library functions [58]⁠. Different datasets were created for each coding sequence (CDS) and sequences with unknown nucleotide, frameshift mutations or premature stop codons were excluded from further analysis.

### Viral genome composition analysis

For each sequence, the following statistics were obtained: content of each nucleotide, total GC content (GC) and in codon positions 1 (GC1), 2 (GC2) and 3 (GC3).

The dinucleotide odds ratio (*Rho)* was computed for each dinucleotide pair using the R library *seqinr* [59]⁠. The *Rho* represents the frequency of dinucleotide (xy) divided by the product of frequencies of nucleotide (x) and nucleotide (y) and should thus be equal to 1.00 when dinucleotide (xy) is formed by chance. Since dinucleotide frequency can be biased by the protein primary structure (i.e. amino acid sequence) and codon usage bias of these genes, a Z-score was calculated normalizing the observed *Rho* by its expectation and variance estimated performing a random sequence generation, which allowed to consistently evaluate the degree of over- or underrepresentation and its statistical significance. Particularly, the selected models generate random sequence by shuffling of synonymous codons, without affecting the codon usage bias and the protein structure. For each sequence, a total of 1000 simulated sequences were generated for dinucleotide pair.

### Relative synonymous codon usage (RSCU) and effective number of codons (Nc)

The RSCU was calculated using the *seqinr* package in R. This statistic, indicative of codon bias, is calculated based on the count of a particular codon, relative to the number of times that the codon would be observed assuming a uniform synonymous codon usage. Consequently, in absence of any codon bias a value close to 1 is expected, while synonymous codons with values lower than 0.6 or greater than 1.6 are classified as under or over-represented, respectively [28, 60].

The Nc values were calculated using the http://agnigarh.tezu.ernet.in/~ssankar/cub.php website [61]⁠. This summary statistic describes the total number of different codons used in a sequence and can thus range between 21 (only one codon used for each amino-acid) and 60 (all synonymous codons are uniformly used) [62]⁠. A second parameter, the Nc’ statistic, also ranging between 21 and 60, was calculated to account for the genome composition effect on codon bias [15, 63]⁠. Obtained Nc and Nc’ values were plotted against the GC3 content of the relative sequence and compared with the expected Nc distribution under the assumption that it is determined only by GC3 content.

### Neutrality plot, general average hydropathicity (gravy) and aromaticity (aroma) indices

A linear regression was calculated between the GC content in the first two codon positions (GC12) of each sequence and the respective GC3 content.

This analysis evaluated the influence of mutational pressure and natural selection on codon usage patterns. The presence of a statistical association and a regression coefficient close to 1 are indicative of mutational bias being the predominant force shaping codon bias patterns.

On the contrary, a regression slope approximating 0 suggests the presence of selective pressure acting on and shaping the codon bias evolution. In this sense, the regression coefficient can be interpreted as a quantitative measure of the mutation-selection equilibrium [64–66]⁠.

Gravy and Aroma indices were calculated using the Peptides [67] package in R. Briefly, the Gravy value is the sum of hydropathy values of all amino acids in a sequence divided by the number of residues, while the Aroma value is the frequency of aromatic amino acids in a given amino acid sequence.

### Principal component analysis (PCA)

A principal component analysis was performed independently on the dinucleotide Z-score and RSCU of all genes, after centering and scaling, using the *prcomp* function of the *stats* library in R [68]. Loadings and eigenvalues associated to each principal component (PC) were evaluated using the same library.

### Codon adaptation index (CAI) calculation

CAI is a summary value (ranging from 0 to 1) that describes the codon usage of a gene relative to the codon usage of a reference set of genes, defining as translationally optimal codons those frequently present in highly expressed genes. It is therefore commonly used to predict the gene expression level based on its coding sequence. In this particular scenario, the CAI value was used to identify the degree of different viral proteins adaptation to the tissue-specific translational machinery. To this purpose, all chicken CDSs were downloaded from Genbank (GCF_000002315.6). Gene expression profiles of different tissues were downloaded from Chickspress [69]⁠. Particularly, lung, kidney and reproductive tract were selected as IBV “target” tissues, while liver and proventriculus were included as “non-target” and “marginal target” tissues, respectively. These were selected as controls representative of tissue with similar features (i.e. parenchymatous or epithelial tissues) known to be irrelevant IBV replication sites. Adipose tissue was also included as representative of a non-target tissue with remarkably different biological features. A collection of tissue-specific highly expressed genes was obtained selecting those whose expression level was in the higher 25 percentile of the considered tissue. The relative CDSs were selected, manually cured (i.e. those with unknown bases or incomplete sequences were removed) and used to calculate the tissue-specific relative adaptiveness of each codon, which was in turn used to calculate the CAI of different proteins for each IBV strain using the *seqinr* [59]⁠ package in R. All images of the present manuscript were drawn using the *ggplot2* library [33]⁠.

## Supplementary Information

**Additional file 1. **Summary of different genome composition and codon bias statistics calculated for each IBV protein. The *P*-value refers to the presence of a significant difference in the mean value of the considered statistic among proteins.

**Additional file 2.** Density plot representing the distribution of nucleotide composition for different IBV coding regions.

**Additional file 3.** Mean and and 95% confidence intervals of Rho statistic calculated for each gene−dinucleotide pair. Structural, non − structural and accessory proteins have been color−coded. Dahed lines represent the cut−offs defined by Karling et al., 1998.

**Additional file 4.** Loadings associated to the RSCU of each codon. When the CpG pair was present in the codon, it has been highlighted in blue.

**Additional file 5.** Scatterplot reporting the relationship between Nc and Nc’ and GC3 content of IBV coding regions. Structural, non − structural and accessory proteins have been color−coded. The line representing the expected Nc values, which would result from GC composition being the only factor influencing the codon usage bias, has been superimposed.

**Additional file 6. **Table reporting the regression coefficient between Gravy or Aroma indexes and the first 2 principal components (PC1 and PC2) of the principal component analyses (PCAs) based on Z-score and RSCU. Regression coefficients have been calculated for different genes, independently. * indicates statistical significance (*p* < 0.05).

**Additional file 7.** Relationship between gene length and total CG count. Two regression lines representative of pp1a, pp1ab and S coding regions (in blue) and another for the remaining proteins (in black) have been superimposed.

## Data Availability

All used IBV sequences are freely available in GenBank (https://www.ncbi.nlm.nih.gov/sites/myncbi/giovanni.franzo.1/collections/59873661/public/), while chicken CDS are available under the genome assembly accession number GCF_000002315.6.

## Abbreviations

IBV: Infectious bronchitis virus
ORF: Open reading frame
PCA: Principal Component Analysis
RSCU: Relative synonymous codon usage
Nc: Effective number of codon
CAI: Codon Adaptation Index
CDS: Coding DNA sequence
TLR: Toll-like receptor
RIG-I: Retinoic acid-inducible gene I
MDA5: Melanoma differentiation-associated protein 5
