# Supplementary material for: Effect of genome composition and codon bias on infectious bronchitis virus evolution and adaptation to target tissues
Source: BMC Genomics. 2021 Apr 7;22:244. doi: 10.1186/s12864-021-07559-5 (PMC8025453; doi:10.1186/s12864-021-07559-5)
Supplement: Supplementary file 1 — Additional file 1. Summary of different genome composition and codon bias statistics calculated for each IBV protein. The P-value refers to the presence of a significant difference in the mean value of the considered statistic among proteins. [file 12864_2021_7559_MOESM1_ESM.docx]

**Supplementary table 1:** Summary of different genome composition and codon bias statistics calculated for each IBV protein. The P-value refers to the presence of a significant difference in the mean value of the considered statistic among proteins.

|  | **1a** | **1ab** | **3a** | **3b** | **5a** | **5b** | **E** | **M** | **N** | **Spike** | **P-value** |
| --- | --- | --- | --- | --- | --- | --- | --- | --- | --- | --- | --- |
| **A** |  |  |  |  |  |  |  |  |  |  |  |
| Minimum | 0.278 | 0.284 | 0.287 | 0.297 | 0.242 | 0.247 | 0.290 | 0.276 | 0.293 | 0.281 |  |
| Maximum | 0.287 | 0.291 | 0.385 | 0.386 | 0.318 | 0.369 | 0.346 | 0.302 | 0.324 | 0.300 |  |
| Median (IQR) | 0.28 (0.28, 0.28) | 0.29 (0.29, 0.29) | 0.31 (0.31, 0.32) | 0.35 (0.33, 0.36) | 0.27 (0.26, 0.29) | 0.34 (0.34, 0.35) | 0.32 (0.31, 0.32) | 0.29 (0.29, 0.29) | 0.31 (0.31, 0.31) | 0.29 (0.29, 0.29) |  |
| Mean ± sd | 0.283 ± 0.001 | 0.288 ± 0.001 | 0.316 ± 0.016 | 0.347 ± 0.017 | 0.275 ± 0.013 | 0.343 ± 0.008 | 0.318 ± 0.007 | 0.291 ± 0.004 | 0.310 ± 0.005 | 0.287 ± 0.003 | *P* < 0.0001 |
| **C** |  |  |  |  |  |  |  |  |  |  |  |
| Minimum | 0.152 | 0.156 | 0.127 | 0.090 | 0.141 | 0.166 | 0.123 | 0.162 | 0.186 | 0.149 |  |
| Maximum | 0.161 | 0.163 | 0.241 | 0.149 | 0.197 | 0.217 | 0.207 | 0.192 | 0.209 | 0.170 |  |
| Median (IQR) | 0.16 (0.16, 0.16) | 0.16 (0.16, 0.16) | 0.20 (0.18, 0.22) | 0.11 (0.11, 0.12) | 0.17 (0.16, 0.18) | 0.20 (0.19, 0.20) | 0.16 (0.15, 0.16) | 0.18 (0.17, 0.19) | 0.20 (0.19, 0.20) | 0.16 (0.16, 0.17) |  |
| Mean ± sd | 0.157 ± 0.002 | 0.160 ± 0.001 | 0.198 ± 0.023 | 0.115 ± 0.010 | 0.168 ± 0.013 | 0.198 ± 0.007 | 0.154 ± 0.013 | 0.179 ± 0.009 | 0.195 ± 0.005 | 0.160 ± 0.004 | *P* < 0.0001 |
| **G** |  |  |  |  |  |  |  |  |  |  |  |
| Minimum | 0.222 | 0.217 | 0.092 | 0.172 | 0.152 | 0.166 | 0.173 | 0.204 | 0.250 | 0.187 |  |
| Maximum | 0.230 | 0.223 | 0.266 | 0.246 | 0.237 | 0.241 | 0.241 | 0.229 | 0.274 | 0.203 |  |
| Median (IQR) | 0.23 (0.22, 0.23) | 0.22 (0.22, 0.22) | 0.14 (0.13, 0.14) | 0.19 (0.19, 0.21) | 0.22 (0.21, 0.22) | 0.22 (0.22, 0.23) | 0.21 (0.21, 0.22) | 0.21 (0.21, 0.22) | 0.26 (0.26, 0.27) | 0.20 (0.19, 0.20) |  |
| Mean ± sd | 0.225 ± 0.002 | 0.220 ± 0.001 | 0.141 ± 0.024 | 0.198 ± 0.012 | 0.216 ± 0.011 | 0.223 ± 0.008 | 0.214 ± 0.007 | 0.214 ± 0.004 | 0.264 ± 0.004 | 0.197 ± 0.004 | *P* < 0.0001 |
| **T** |  |  |  |  |  |  |  |  |  |  |  |
| Minimum | 0.331 | 0.329 | 0.277 | 0.287 | 0.313 | 0.221 | 0.275 | 0.301 | 0.218 | 0.346 |  |
| Maximum | 0.340 | 0.337 | 0.397 | 0.370 | 0.379 | 0.421 | 0.346 | 0.342 | 0.246 | 0.362 |  |
| Median (IQR) | 0.33 (0.33, 0.34) | 0.33 (0.33, 0.33) | 0.34 (0.33, 0.36) | 0.34 (0.33, 0.34) | 0.34 (0.33, 0.35) | 0.24 (0.23, 0.24) | 0.31 (0.31, 0.32) | 0.31 (0.31, 0.32) | 0.23 (0.23, 0.23) | 0.36 (0.35, 0.36) |  |
| Mean ± sd | 0.334 ± 0.002 | 0.332 ± 0.002 | 0.345 ± 0.021 | 0.339 ± 0.011 | 0.341 ± 0.011 | 0.236 ± 0.014 | 0.314 ± 0.012 | 0.316 ± 0.009 | 0.231 ± 0.005 | 0.355 ± 0.003 | *P* < 0.0001 |
| **GC** |  |  |  |  |  |  |  |  |  |  |  |
| Minimum | 0.377 | 0.376 | 0.276 | 0.286 | 0.338 | 0.332 | 0.330 | 0.372 | 0.445 | 0.346 |  |
| Maximum | 0.388 | 0.383 | 0.416 | 0.359 | 0.429 | 0.442 | 0.404 | 0.407 | 0.472 | 0.367 |  |
| Median (IQR) | 0.38 (0.38, 0.38) | 0.38 (0.38, 0.38) | 0.34 (0.33, 0.34) | 0.31 (0.30, 0.32) | 0.38 (0.38, 0.39) | 0.42 (0.41, 0.43) | 0.37 (0.36, 0.38) | 0.40 (0.39, 0.40) | 0.46 (0.46, 0.46) | 0.36 (0.36, 0.36) |  |
| Mean ± sd | 0.382 ± 0.002 | 0.380 ± 0.001 | 0.339 ± 0.019 | 0.314 ± 0.018 | 0.383 ± 0.016 | 0.420 ± 0.012 | 0.368 ± 0.015 | 0.393 ± 0.009 | 0.459 ± 0.005 | 0.357 ± 0.004 | *P* < 0.0001 |
| **GC1** |  |  |  |  |  |  |  |  |  |  |  |
| Minimum | 0.488 | 0.481 | 0.241 | 0.400 | 0.348 | 0.470 | 0.435 | 0.412 | 0.554 | 0.415 |  |
| Maximum | 0.501 | 0.490 | 0.483 | 0.523 | 0.485 | 0.530 | 0.528 | 0.465 | 0.590 | 0.445 |  |
| Median (IQR) | 0.49 (0.49, 0.49) | 0.49 (0.48, 0.49) | 0.41 (0.41, 0.43) | 0.45 (0.43, 0.48) | 0.41 (0.39, 0.42) | 0.49 (0.49, 0.51) | 0.49 (0.46, 0.51) | 0.44 (0.43, 0.45) | 0.57 (0.57, 0.57) | 0.44 (0.43, 0.44) |  |
| Mean ± sd | 0.493 ± 0.002 | 0.486 ± 0.002 | 0.413 ± 0.040 | 0.456 ± 0.024 | 0.411 ± 0.024 | 0.498 ± 0.011 | 0.484 ± 0.024 | 0.439 ± 0.010 | 0.570 ± 0.007 | 0.435 ± 0.005 | *P* < 0.0001 |
| **GC2** |  |  |  |  |  |  |  |  |  |  |  |
| Minimum | 0.364 | 0.362 | 0.226 | 0.206 | 0.333 | 0.280 | 0.324 | 0.381 | 0.468 | 0.389 |  |
| Maximum | 0.372 | 0.369 | 0.431 | 0.369 | 0.470 | 0.470 | 0.389 | 0.434 | 0.502 | 0.413 |  |
| Median (IQR) | 0.37 (0.37, 0.37) | 0.37 (0.36, 0.37) | 0.33 (0.28, 0.36) | 0.29 (0.28, 0.29) | 0.42 (0.39, 0.42) | 0.43 (0.42, 0.45) | 0.34 (0.34, 0.35) | 0.40 (0.40, 0.41) | 0.49 (0.49, 0.49) | 0.40 (0.40, 0.40) |  |
| Mean ± sd | 0.368 ± 0.001 | 0.365 ± 0.001 | 0.325 ± 0.049 | 0.283 ± 0.024 | 0.412 ± 0.021 | 0.428 ± 0.022 | 0.348 ± 0.012 | 0.403 ± 0.008 | 0.489 ± 0.005 | 0.402 ± 0.004 | *P* < 0.0001 |
| **GC3** |  |  |  |  |  |  |  |  |  |  |  |
| Minimum | 0.275 | 0.280 | 0.172 | 0.123 | 0.273 | 0.220 | 0.213 | 0.292 | 0.288 | 0.210 |  |
| Maximum | 0.297 | 0.297 | 0.456 | 0.297 | 0.409 | 0.386 | 0.370 | 0.376 | 0.351 | 0.264 |  |
| Median (IQR) | 0.29 (0.28, 0.29) | 0.29 (0.29, 0.29) | 0.28 (0.26, 0.29) | 0.20 (0.17, 0.23) | 0.32 (0.30, 0.35) | 0.34 (0.33, 0.34) | 0.27 (0.26, 0.28) | 0.34 (0.32, 0.35) | 0.31 (0.31, 0.33) | 0.24 (0.23, 0.24) |  |
| Mean ± sd | 0.285 ± 0.004 | 0.289 ± 0.003 | 0.279 ± 0.042 | 0.202 ± 0.040 | 0.327 ± 0.027 | 0.336 ± 0.016 | 0.271 ± 0.030 | 0.337 ± 0.018 | 0.318 ± 0.014 | 0.235 ± 0.008 | *P* < 0.0001 |
| **Nc** |  |  |  |  |  |  |  |  |  |  |  |
| Minimum | 44.67 | 45.43 | 24.82 | 22.96 | 28.08 | 30.50 | 32.10 | 40.84 | 42.45 | 40.41 |  |
| Maximum | 46.87 | 46.80 | 32.21 | 31.45 | 38.34 | 40.69 | 43.51 | 46.88 | 48.50 | 44.32 |  |
| Median (IQR) | 45.47 (45.29, 45.68) | 46.04 (45.92, 46.19) | 28.79 (28.05, 29.82) | 28.01 (25.69, 29.29) | 31.80 (30.57, 33.19) | 37.57 (37.20, 38.20) | 35.98 (35.14, 37.27) | 44.39 (42.89, 45.05) | 45.53 (44.78, 47.03) | 41.92 (41.33, 42.16) |  |
| Mean ± sd | 45.461 ± 0.341 | 46.034 ± 0.223 | 28.850 ± 1.469 | 27.603 ± 1.813 | 31.760 ± 1.765 | 37.700 ± 1.025 | 36.188 ± 1.751 | 43.907 ± 1.495 | 45.796 ± 1.425 | 41.881 ± 0.720 | *P* < 0.0001 |
| **Nc’** |  |  |  |  |  |  |  |  |  |  |  |
| Minimum | 55.66 | 55.97 | 28.55 | 30.10 | 29.79 | 35.16 | 36.00 | 44.39 | 47.15 | 53.90 |  |
| Maximum | 57.14 | 57.36 | 37.29 | 40.93 | 40.22 | 43.64 | 48.43 | 53.64 | 54.48 | 56.69 |  |
| Median (IQR) | 56.37 (56.15, 56.59) | 56.61 (56.42, 56.76) | 33.36 (30.92, 34.39) | 36.52 (35.14, 38.78) | 34.97 (33.51, 35.33) | 40.67 (39.97, 41.16) | 41.46 (40.89, 42.51) | 47.53 (46.98, 48.10) | 51.93 (51.20, 52.71) | 55.30 (54.96, 55.68) |  |
| Mean ± sd | 56.377 ± 0.277 | 56.568 ± 0.278 | 32.936 ± 2.149 | 36.753 ± 2.247 | 34.396 ± 1.920 | 40.611 ± 1.141 | 41.851 ± 1.501 | 47.603 ± 1.049 | 51.865 ± 1.061 | 55.343 ± 0.532 | *P* < 0.0001 |
| **CAI: Lung** |  |  |  |  |  |  |  |  |  |  |  |
| Minimum | 0.701 | 0.705 | 0.501 | 0.638 | 0.523 | 0.603 | 0.638 | 0.685 | 0.752 | 0.689 |  |
| Maximum | 0.713 | 0.713 | 0.711 | 0.723 | 0.661 | 0.721 | 0.711 | 0.722 | 0.789 | 0.707 |  |
| Median (IQR) | 0.71 (0.71, 0.71) | 0.71 (0.71, 0.71) | 0.55 (0.54, 0.58) | 0.69 (0.68, 0.71) | 0.57 (0.55, 0.58) | 0.70 (0.69, 0.70) | 0.66 (0.65, 0.67) | 0.70 (0.69, 0.71) | 0.77 (0.76, 0.77) | 0.70 (0.69, 0.70) |  |
| Mean ± sd | 0.707 ± 0.002 | 0.708 ± 0.002 | 0.568 ± 0.039 | 0.692 ± 0.017 | 0.570 ± 0.024 | 0.695 ± 0.013 | 0.663 ± 0.013 | 0.703 ± 0.010 | 0.768 ± 0.007 | 0.696 ± 0.003 | *P* < 0.0001 |

| **CAI: Kidney** |  |  |  |  |  |  |  |  |  |  |  |
| --- | --- | --- | --- | --- | --- | --- | --- | --- | --- | --- | --- |
| Minimum | 0.693 | 0.697 | 0.491 | 0.626 | 0.518 | 0.593 | 0.629 | 0.677 | 0.747 | 0.680 |  |
| Maximum | 0.705 | 0.705 | 0.704 | 0.712 | 0.652 | 0.715 | 0.702 | 0.715 | 0.785 | 0.698 |  |
| Median (IQR) | 0.70 (0.70, 0.70) | 0.70 (0.70, 0.70) | 0.54 (0.53, 0.57) | 0.68 (0.67, 0.70) | 0.56 (0.55, 0.57) | 0.69 (0.68, 0.70) | 0.65 (0.64, 0.66) | 0.69 (0.69, 0.70) | 0.76 (0.76, 0.77) | 0.69 (0.69, 0.69) |  |
| Mean ± sd | 0.699 ± 0.002 | 0.700 ± 0.002 | 0.559 ± 0.039 | 0.681 ± 0.018 | 0.563 ± 0.024 | 0.688 ± 0.013 | 0.654 ± 0.013 | 0.695 ± 0.010 | 0.764 ± 0.007 | 0.688 ± 0.003 | *P* < 0.0001 |
| **CAI: Ovary** |  |  |  |  |  |  |  |  |  |  |  |
| Minimum | 0.708 | 0.712 | 0.510 | 0.649 | 0.529 | 0.612 | 0.646 | 0.691 | 0.755 | 0.696 |  |
| Maximum | 0.720 | 0.720 | 0.717 | 0.732 | 0.669 | 0.725 | 0.718 | 0.728 | 0.792 | 0.715 |  |
| Median (IQR) | 0.71 (0.71, 0.72) | 0.72 (0.71, 0.72) | 0.56 (0.55, 0.59) | 0.70 (0.69, 0.72) | 0.58 (0.56, 0.59) | 0.70 (0.69, 0.71) | 0.67 (0.66, 0.68) | 0.71 (0.70, 0.72) | 0.77 (0.77, 0.78) | 0.70 (0.70, 0.71) |  |
| Mean ± sd | 0.715 ± 0.002 | 0.716 ± 0.002 | 0.577 ± 0.039 | 0.702 ± 0.017 | 0.577 ± 0.024 | 0.700 ± 0.013 | 0.670 ± 0.013 | 0.709 ± 0.009 | 0.771 ± 0.007 | 0.704 ± 0.003 | *P* < 0.0001 |
| **CAI: Adipose** |  |  |  |  |  |  |  |  |  |  |  |
| Minimum | 0.683 | 0.688 | 0.480 | 0.613 | 0.512 | 0.582 | 0.619 | 0.668 | 0.742 | 0.670 |  |
| Maximum | 0.696 | 0.696 | 0.697 | 0.701 | 0.642 | 0.708 | 0.693 | 0.707 | 0.780 | 0.688 |  |
| Median (IQR) | 0.69 (0.69, 0.69) | 0.69 (0.69, 0.69) | 0.53 (0.53, 0.56) | 0.67 (0.66, 0.69) | 0.56 (0.54, 0.57) | 0.68 (0.68, 0.69) | 0.64 (0.63, 0.65) | 0.68 (0.68, 0.70) | 0.76 (0.75, 0.76) | 0.68 (0.68, 0.68) |  |
| Mean ± sd | 0.690 ± 0.002 | 0.691 ± 0.002 | 0.548 ± 0.039 | 0.669 ± 0.018 | 0.556 ± 0.023 | 0.681 ± 0.013 | 0.645 ± 0.013 | 0.686 ± 0.010 | 0.759 ± 0.007 | 0.678 ± 0.003 | *P* < 0.0001 |
| **CAI: Liver** |  |  |  |  |  |  |  |  |  |  |  |
| Minimum | 0.682 | 0.686 | 0.478 | 0.611 | 0.511 | 0.580 | 0.618 | 0.668 | 0.742 | 0.668 |  |
| Maximum | 0.695 | 0.695 | 0.695 | 0.699 | 0.640 | 0.708 | 0.691 | 0.706 | 0.779 | 0.687 |  |
| Median (IQR) | 0.69 (0.69, 0.69) | 0.69 (0.69, 0.69) | 0.53 (0.52, 0.56) | 0.67 (0.66, 0.68) | 0.56 (0.54, 0.57) | 0.68 (0.68, 0.69) | 0.64 (0.63, 0.65) | 0.68 (0.68, 0.70) | 0.76 (0.75, 0.76) | 0.68 (0.67, 0.68) |  |
| Mean ± sd | 0.689 ± 0.002 | 0.690 ± 0.002 | 0.547 ± 0.039 | 0.667 ± 0.018 | 0.555 ± 0.023 | 0.681 ± 0.013 | 0.644 ± 0.013 | 0.685 ± 0.010 | 0.759 ± 0.007 | 0.676 ± 0.003 | *P* < 0.0001 |
| **CAI: Proventriculus** |  |  |  |  |  |  |  |  |  |  |  |
| Minimum | 0.679 | 0.683 | 0.475 | 0.607 | 0.509 | 0.577 | 0.614 | 0.665 | 0.739 | 0.665 |  |
| Maximum | 0.692 | 0.692 | 0.693 | 0.695 | 0.637 | 0.706 | 0.688 | 0.703 | 0.777 | 0.683 |  |
| Median (IQR) | 0.69 (0.68, 0.69) | 0.69 (0.69, 0.69) | 0.53 (0.52, 0.55) | 0.66 (0.65, 0.68) | 0.55 (0.54, 0.56) | 0.68 (0.67, 0.69) | 0.64 (0.63, 0.65) | 0.68 (0.67, 0.69) | 0.75 (0.75, 0.76) | 0.67 (0.67, 0.67) |  |
| Mean ± sd | 0.686 ± 0.002 | 0.687 ± 0.002 | 0.544 ± 0.039 | 0.662 ± 0.018 | 0.553 ± 0.023 | 0.679 ± 0.013 | 0.640 ± 0.013 | 0.683 ± 0.010 | 0.756 ± 0.007 | 0.673 ± 0.003 | *P* < 0.0001 |
