# Supplementary figures and images for: Effect of genome composition and codon bias on infectious bronchitis virus evolution and adaptation to target tissues
Source: BMC Genomics. 2021 Apr 7;22:244. doi: 10.1186/s12864-021-07559-5 (PMC8025453; doi:10.1186/s12864-021-07559-5)

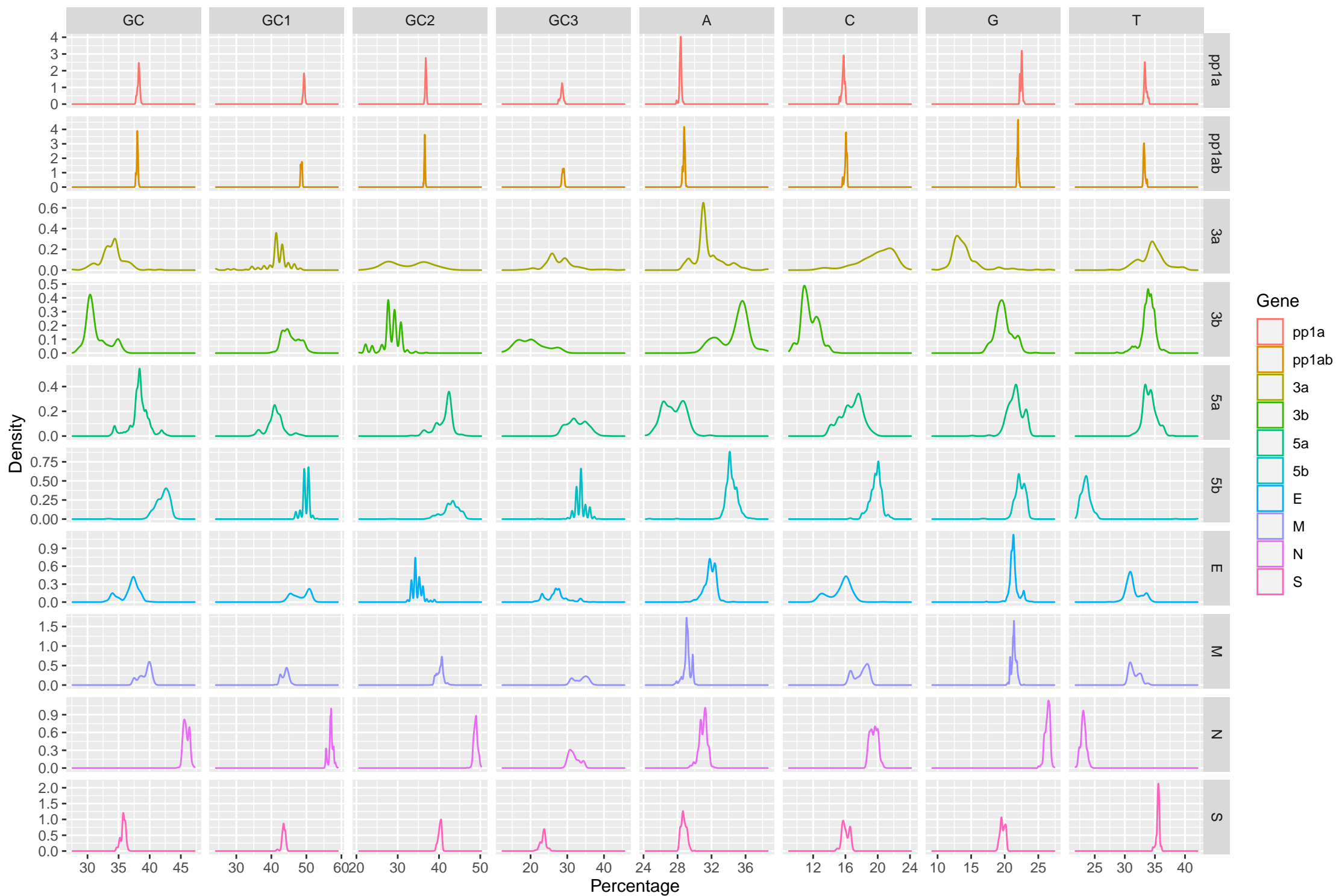

Supplement: Supplementary file 2 — Additional file 2. Density plot representing the distribution of nucleotide composition for different IBV coding regions. [file 12864_2021_7559_MOESM2_ESM.pdf]

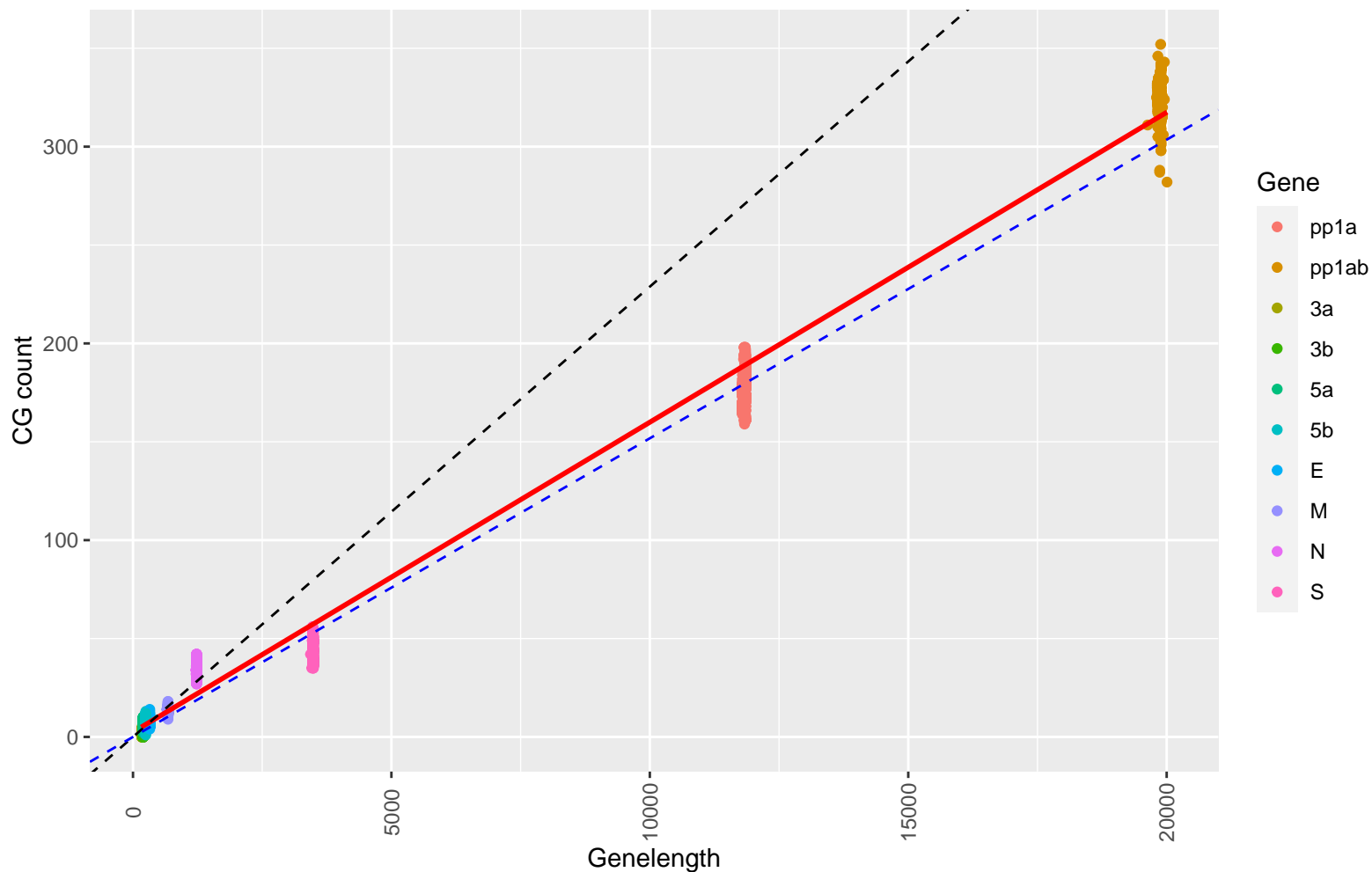

Supplement: Supplementary file 7 — Additional file 7. Relationship between gene length and total CG count. Two regression lines representative of pp1a, pp1ab and S coding regions (in blue) and another for the remaining proteins (in black) have been superimposed. [file 12864_2021_7559_MOESM7_ESM.pdf]
