# Supplementary material for: Effect of genome composition and codon bias on infectious bronchitis virus evolution and adaptation to target tissues
Source: BMC Genomics. 2021 Apr 7;22:244. doi: 10.1186/s12864-021-07559-5 (PMC8025453; doi:10.1186/s12864-021-07559-5)
Supplement: Supplementary file 3 — Additional file 3. Mean and and 95% confidence intervals of Rho statistic calculated for each gene−dinucleotide pair. Structural, non − structural and accessory proteins have been color−coded. Dahed lines represent the cut−offs defined by Karling et al., 1998. [file 12864_2021_7559_MOESM3_ESM.pdf]

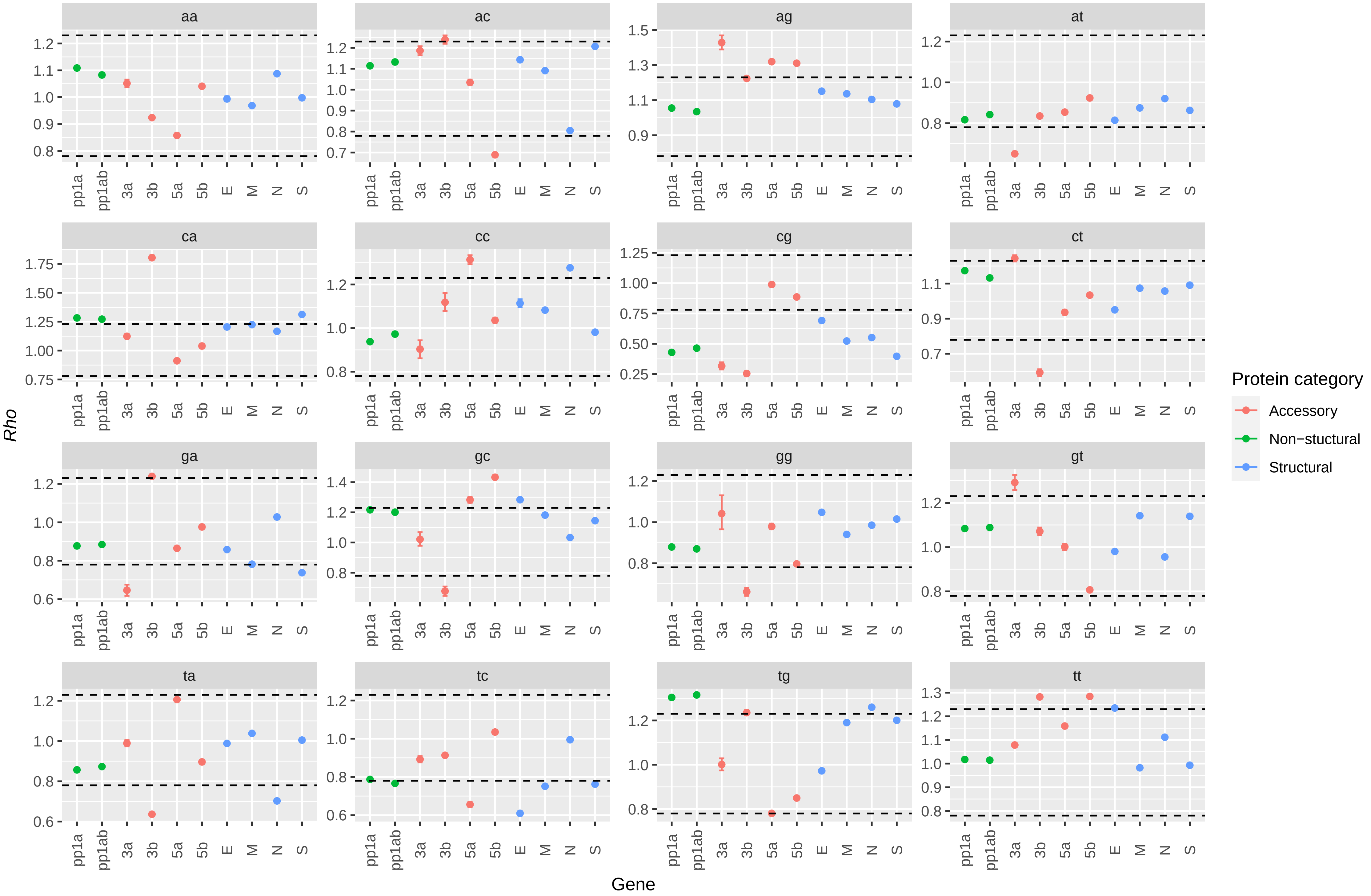

Mean and and 95% confidence intervals of Rho statistic calculated for each gene−dinucleotide pair.
Structural, non−structural and accessory proteins have been color−coded. Dahed lines represent the cut−offs defined by Karling et al., 1998
