# Supplementary material for: Effect of genome composition and codon bias on infectious bronchitis virus evolution and adaptation to target tissues
Source: BMC Genomics. 2021 Apr 7;22:244. doi: 10.1186/s12864-021-07559-5 (PMC8025453; doi:10.1186/s12864-021-07559-5)
Supplement: Supplementary file 4 — Additional file 4. Loadings associated to the RSCU of each codon. When the CpG pair was present in the codon, it has been highlighted in blue. [file 12864_2021_7559_MOESM4_ESM.pdf]

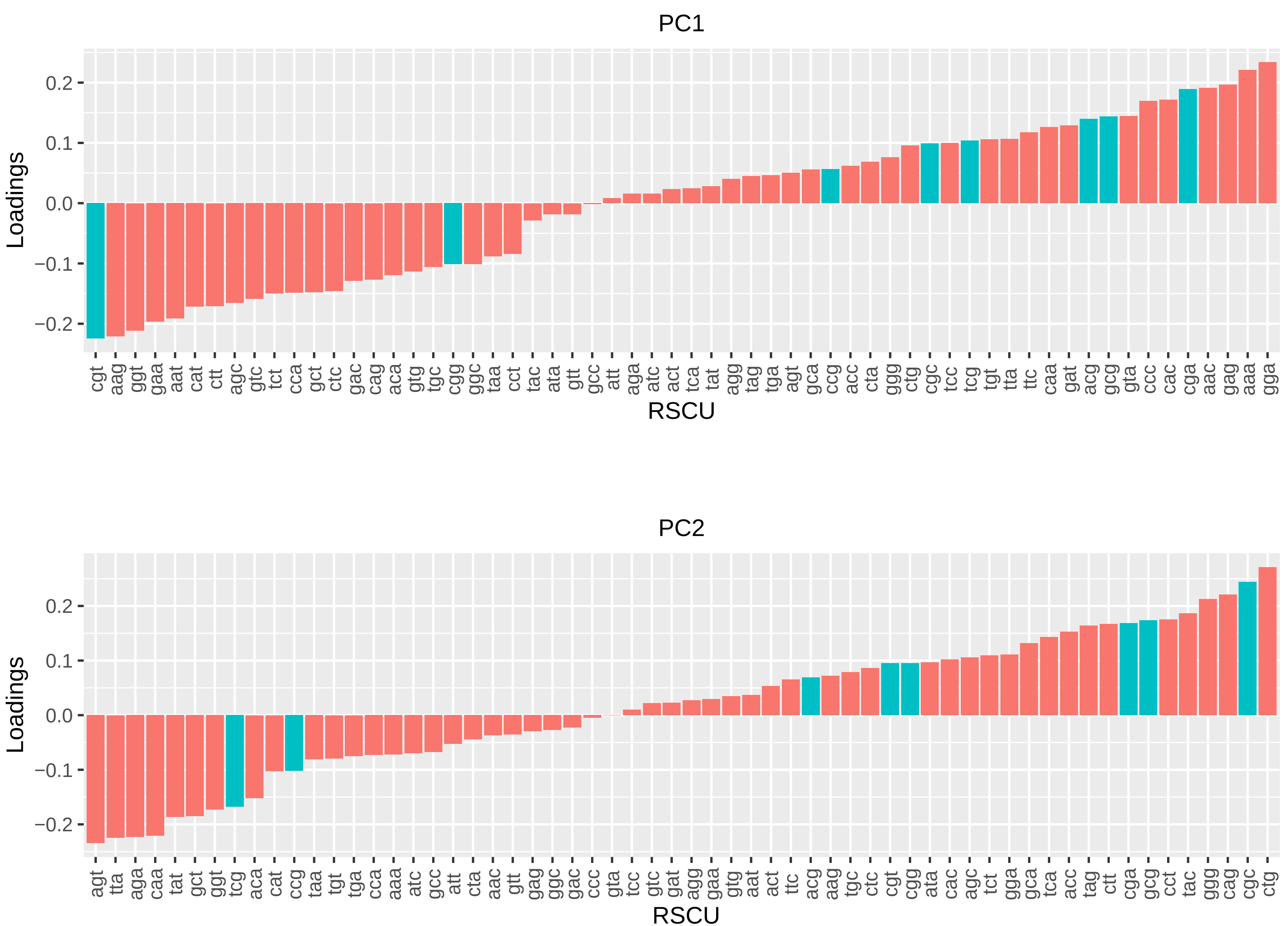

Loadings associated to the RSCU of each codon. When the CpG pair was present in the codon, it has been highlighted in blue

CG No Yes
