# Supplementary material for: Effect of genome composition and codon bias on infectious bronchitis virus evolution and adaptation to target tissues
Source: BMC Genomics. 2021 Apr 7;22:244. doi: 10.1186/s12864-021-07559-5 (PMC8025453; doi:10.1186/s12864-021-07559-5)
Supplement: Supplementary file 5 — Additional file 5. Scatterplot reporting the relationship between Nc and Nc’ and GC3 content of IBV coding regions. Structural, non − structural and accessory proteins have been color−coded. The line representing the expected Nc values, which would result from GC composition being the only factor influencing the codon usage bias, has been superimposed. [file 12864_2021_7559_MOESM5_ESM.pdf]

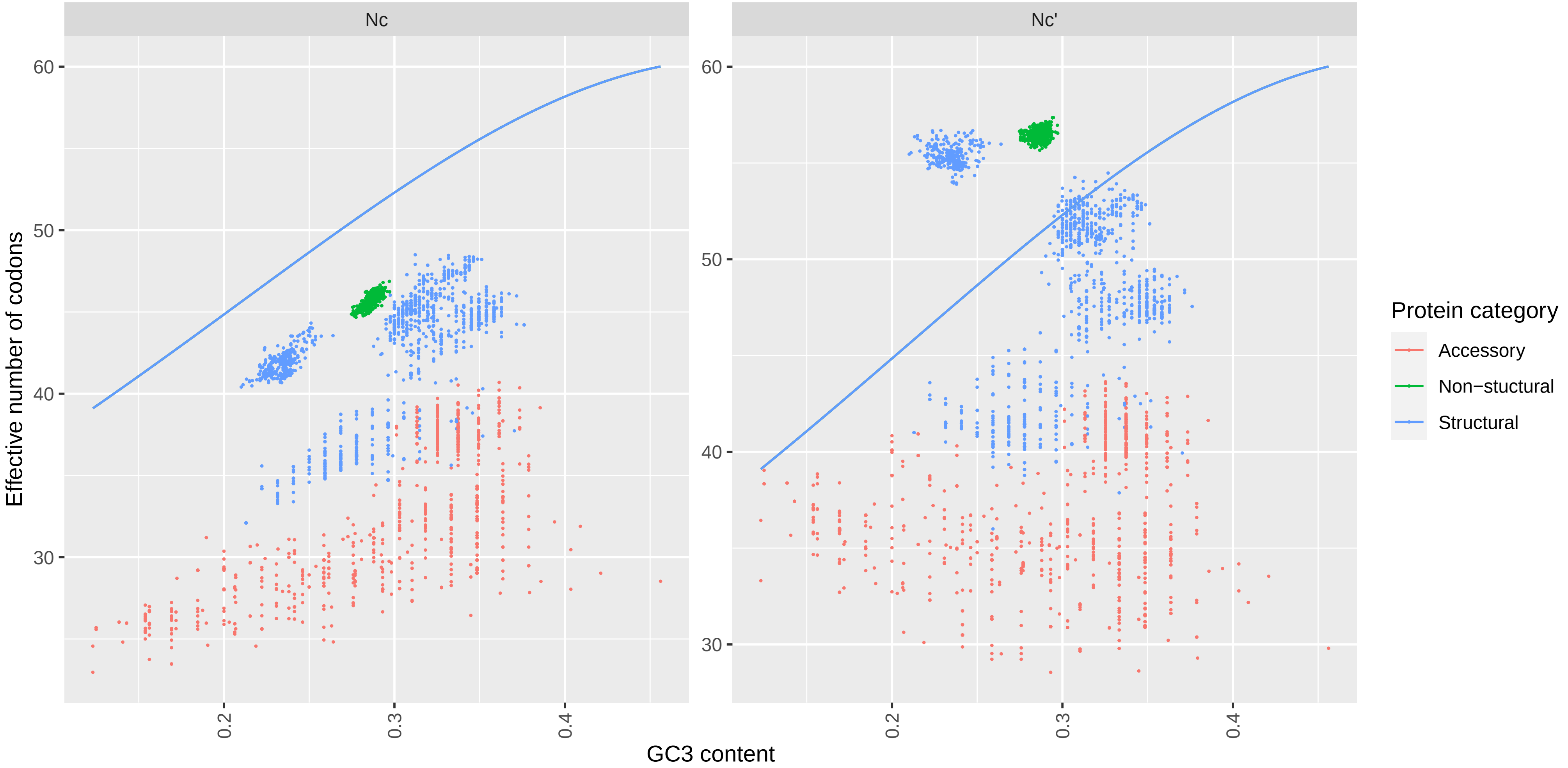

Scatterplot reporting the relationship between Nc and Nc' and GC3 content of IBV coding regions. Structural, non−structural and accessory proteins have been color−coded. The line representing the expected Nc values, which would result from GC composition being the only factor influencing the codon usage bias, has been superimposed.
