# Supplementary material for: Effect of genome composition and codon bias on infectious bronchitis virus evolution and adaptation to target tissues
Source: BMC Genomics. 2021 Apr 7;22:244. doi: 10.1186/s12864-021-07559-5 (PMC8025453; doi:10.1186/s12864-021-07559-5)
Supplement: Supplementary file 6 — Additional file 6. Table reporting the regression coefficient between Gravy or Aroma indexes and the first 2 principal components (PC1 and PC2) of the principal component analyses (PCAs) based on Z-score and RSCU. Regression coefficients have been calculated for different genes, independently. * indicates statistical significance (p < 0.05). [file 12864_2021_7559_MOESM6_ESM.docx]

|  |  | Z-score | | RSCU | |
| --- | --- | --- | --- | --- | --- |
|  |  | PC1 | PC2 | PC1 | PC2 |
| Gravy | 1a | 0.0009 | -0.0055* | -0.0123* | -0.0048 |
|  | 1ab | 0.0322 | -0.0036* | -0.0045* | -0.0146* |
|  | 3a | 0.2749* | -0.0338 | 0.0296* | -0.0495* |
|  | 3b | 0.0021 | -0.0459* | 0.0006 | -0.0080* |
|  | 5a | 0.0017 | 0.0955* | -0.0120* | 0.0073* |
|  | 5b | -0.1911* | 0.1642* | 0.0283* | 0.0557* |
|  | E | -0.0293* | 0.0186* | 0.0032* | -0.0060* |
|  | M | -0.0048 | -0.0032 | 0.0032* | 0.0048* |
|  | N | 0.0386* | 0.0157* | -0.0044* | -0.0016* |
|  | Spike | 0.0122* | 0.0021 | -0.0030 | 0.0188* |
| Aroma | 1a | 0.0236 | -0.0493* | -0.2146* | 0.1859* |
|  | 1ab | 0.0177* | -0.0099* | -0.0258* | 0.1203* |
|  | 3a | -2.8079* | 1.0690* | 0.4201* | 0.0454 |
|  | 3b | 0.2785 | -0.0092 | 0.0259 | -0.0020 |
|  | 5a | -1.8166* | 1.0518* | 0.1088* | 0.2901* |
|  | 5b | -0.4574* | 0.7997* | 0.2235* | 0.3535* |
|  | E | 0.3995* | 0.3550* | 0.1325* | -0.2363* |
|  | M | 0.3026* | -0.1103* | 0.0764* | 0.0515* |
|  | N | -0.0653* | -0.0085* | 0.0266* | -0.0248* |
|  | Spike | -0.1179* | 0.1754* | 0.0120 | -0.1503* |

Additional file 6. Table reporting the regression coefficient between Gravy or Aroma indexes and the first 2 principal components (PC1 and PC2) of the principal component analyses (PCAs) based on Z-score and RSCU. Regression coefficients have been calculated for different genes, independently. * indicates statistical significance (p<0.05).
